# T-cell Lymphoblastic Lymphoma Unveiling As Superior Vena Cava Syndrome in a 19-Year-Old Male

**DOI:** 10.7759/cureus.54729

**Published:** 2024-02-22

**Authors:** Rinkle Gemnani, Keyur Saboo, Rajvardhan Patil, Sunil Kumar, Sourya Acharya

**Affiliations:** 1 Department of Medicine, Jawaharlal Nehru Medical College, Datta Meghe Institute of Higher Education and Research (Deemed to be University), Wardha, IND; 2 Department of Internal Medicine, Jawaharlal Nehru Medical College, Datta Meghe Institute of Higher Education and Research (Deemed to be University), Wardha, IND

**Keywords:** superior vena cava syndrome, non-hodgkin lymphoma, case report, mediastinal mass, t-lymphoblastic lymphoma

## Abstract

Superior vena cava syndrome (SVCS) is a collection of signs and symptoms resulting from superior vena cava obstruction which is either partial or complete. SVCS is a rare clinical entity, often associated with various malignancies. T-cell lymphoblastic lymphoma (T-LBL) primarily of the mediastinum (thymus) is a rare and aggressive non-Hodgkin lymphoma that can lead to SVCS. We discuss the case of a 19-year-old male who arrived at our emergency department with symptoms of cough, breathlessness, and facial puffiness along with swelling in the right anterior mediastinum for two weeks suggestive of acute SVCS. An anterior mediastinal mass was confirmed on a chest X-ray and computed tomography. A biopsy of the mass revealed primary mediastinal (thymic) T-LBL. This case report focuses on the unique presentation of a T-LBL as SVCS in a 19-year-old male. Moreover, it highlights the need for vigilance among healthcare providers in recognizing this atypical complication and underscores the critical importance of early diagnosis and timely intervention.

## Introduction

Superior vena cava syndrome (SVCS) encompasses a collection of clinical signs and symptoms resulting from partial or complete obstruction of the blood flow through the superior vena cava. Clinical features of this syndrome are swelling in the upper body, including the head, neck, arms, and breast, as well as cyanosis, plethora, and dilated subcutaneous veins. This edema can induce functional abnormalities in the larynx or pharynx, contributing to cough, hoarseness, dyspnea, and dysphagia. Nowadays, neoplasm is the most common cause of developing SVCS. The primary factor contributing to the development of SVCS is the rate at which venous restriction first appears. Collateral circulation via the azygous vein and inferior vena cava may arise from slowly progressing superior vena cava lesions, which may lessen or even completely prevent the development of SVCS [1].

SVCS may arise due to the intrinsic interruption of superior vena cava flow owing to thrombus development. Two possible causes of extrinsic compression are benign and malignant [2,3]. About 10% of cases with SVCS are caused by small cell lung cancer, which is the most frequent extrinsic etiology. In addition to non-malignant causes like aortic aneurysms, other extrinsic etiologies of SVCS are lymphoma and metastatic illness [4]. Nevertheless, an uncommon and possibly fatal consequence of non-Hodgkin lymphoma (NHL) is SVCS. Only 2-4% of NHL cases are reported to develop this entity [5]. Improving the prognosis requires prompt and efficient intervention to treat the malignant etiology of SVCS.

Therefore, primary mediastinal (thymic) T-cell lymphoblastic lymphoma (T-LBL) represents a unique subtype of NHL, characterized by aggressive behavior and particular clinicopathologic features. According to the literature, a few instances are first presented with SVCS [5,6]. We report a rare case of biopsy-proven T-LBL which presented as SVCS in a 19-year-old male.

## Case presentation

A 19-year-old male presented to our emergency department with the chief complaint of dry cough associated with dyspnea for one month. The cough was sudden in onset, worsening in the dorsal decubitus position and reduced in the propped-up position associated with dyspnea which intensified with the orthostatic position. The patient also complained of hoarseness of voice for 15 days and facial puffiness for 10 days. For the same complaints, the patient went to a local primary healthcare (PHC) five days before coming to our center where he was prescribed bromhexine syrup two teaspoons twice a day for three days, a tablet of theophylline 400 mg twice a day for three days, and a tablet cetirizine 10 mg once at night for three days but had no relief. On further questioning, the patient had a history of weight loss, around 5 kg in the past two months. He denied any significant past medical or surgical history, and he had no history of previous hospitalization.

**Figure 1:**
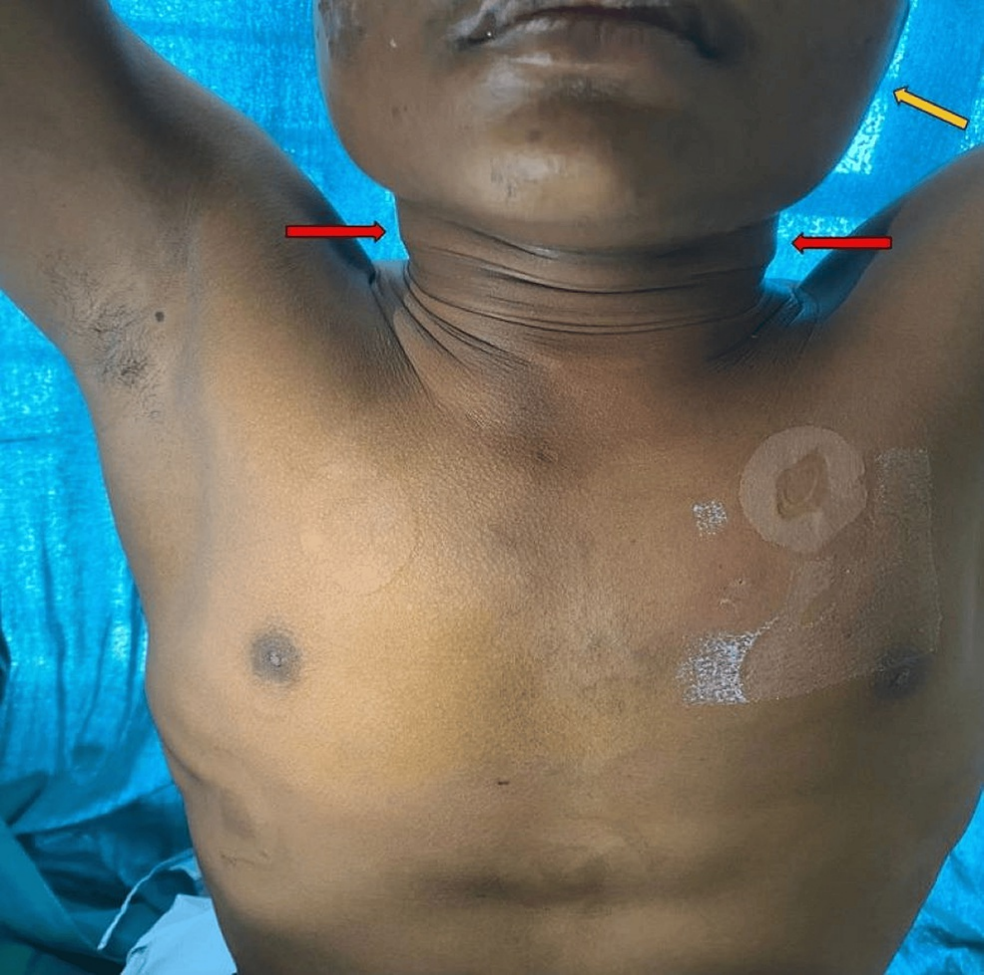
Dilated neck veins (red arrows) and facial puffiness (yellow arrow).

On admission, the patient was moderately built and afebrile to touch with a pulse rate of 90 beats per minute, blood pressure of 110/70 mmHg, and respiratory rate of 30 cycles per minute maintaining a saturation of 96% on room air. On general examination, the patient had pallor, facial puffiness, and dilated veins over the neck and chest, as shown in Figure 1.

**Figure 2:**
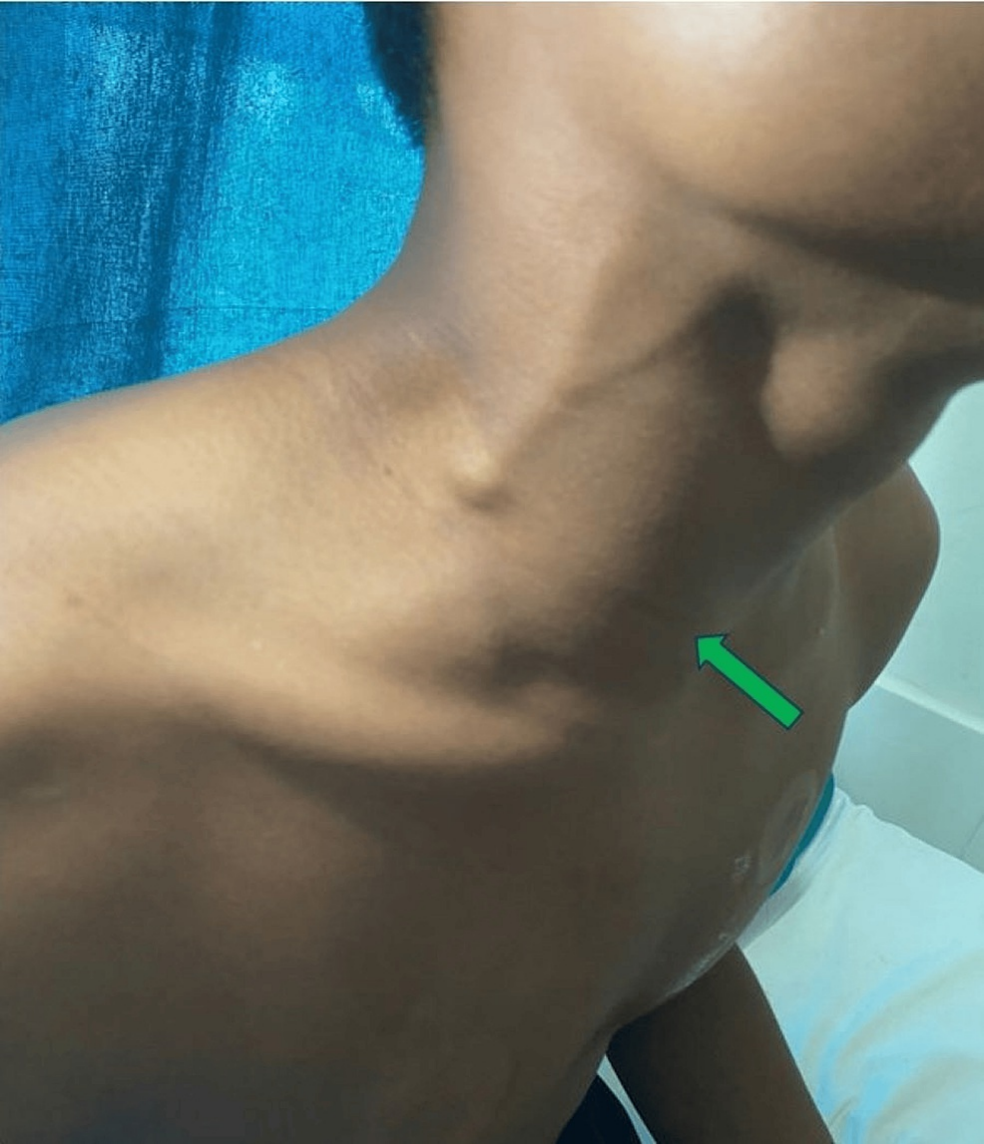
Mass in the neck extending into the infraclavicular space (green arrow).

On palpation, an anterior neck mass measuring 8x5 cm along with a right supraclavicular lymph node measuring about 0.8x0.5 cm was found. The said mass extended from the middle of the neck to the right infraclavicular space as shown in Figure 2.

Bilateral coarse breath sounds were heard on lung field auscultation with occasional wheeze. Air entry was absent at the right inframammary, infrascapular, and infra-axillary areas. The rest of the systemic examination was unremarkable. Blood investigations were within normal limits as shown in Table 1. An electrocardiogram (ECG) revealed sinus rhythm at a rate of 90 beats per minute.

**Table 1:** Laboratory parameter of the patient with reference range. PaO2: partial pressure of arterial oxygen; PaCO2: partial pressure of arterial carbon dioxide

Investigations	Patient value	Reference value
Hemoglobin	12.5 g/dL (low)	13-17 g/dL
Total leukocyte count	6,600/dL (normal)	4,000-11,000/dL
Mean corpuscular volume	82 fL (normal)	83-101 fL
Platelet count	292,000/dL (normal)	150,000-400,000/dL
Urea	20 mg/dL (normal)	19-42 mg/dL
Serum creatinine	0.8 mg/dL (normal)	0.5-1.2 mg/dL
Sodium	143 mmol/L (normal)	137-145 mmol/L
Potassium	4 mmol/l (normal)	3.5-5.1 mmol/L
Albumin	3.8 g/dL (normal)	3.5-5 g/dL
Aspartate aminotransferase	40 U/L (normal)	<50 U/L
Alanine aminotransferase	52 U/L (normal)	17-59 U/L
Total bilirubin	0.8 mg/dL (normal)	0.2-1.3 mg/dL
Calcium	9 mg/dL (normal)	8.5-10 mg/dL
Activated partial thromboplastin time	29.9 (normal)	29.5-31
Prothrombin time	13 (normal)	11.9-13
International normalized ratio	1.01 (normal)	0.9-1.4
PaO2	96 mmHg (normal)	80-100 mmHg
PaCO2	38 mmHg (normal)	35-45 mmHg

**Figure 3:**
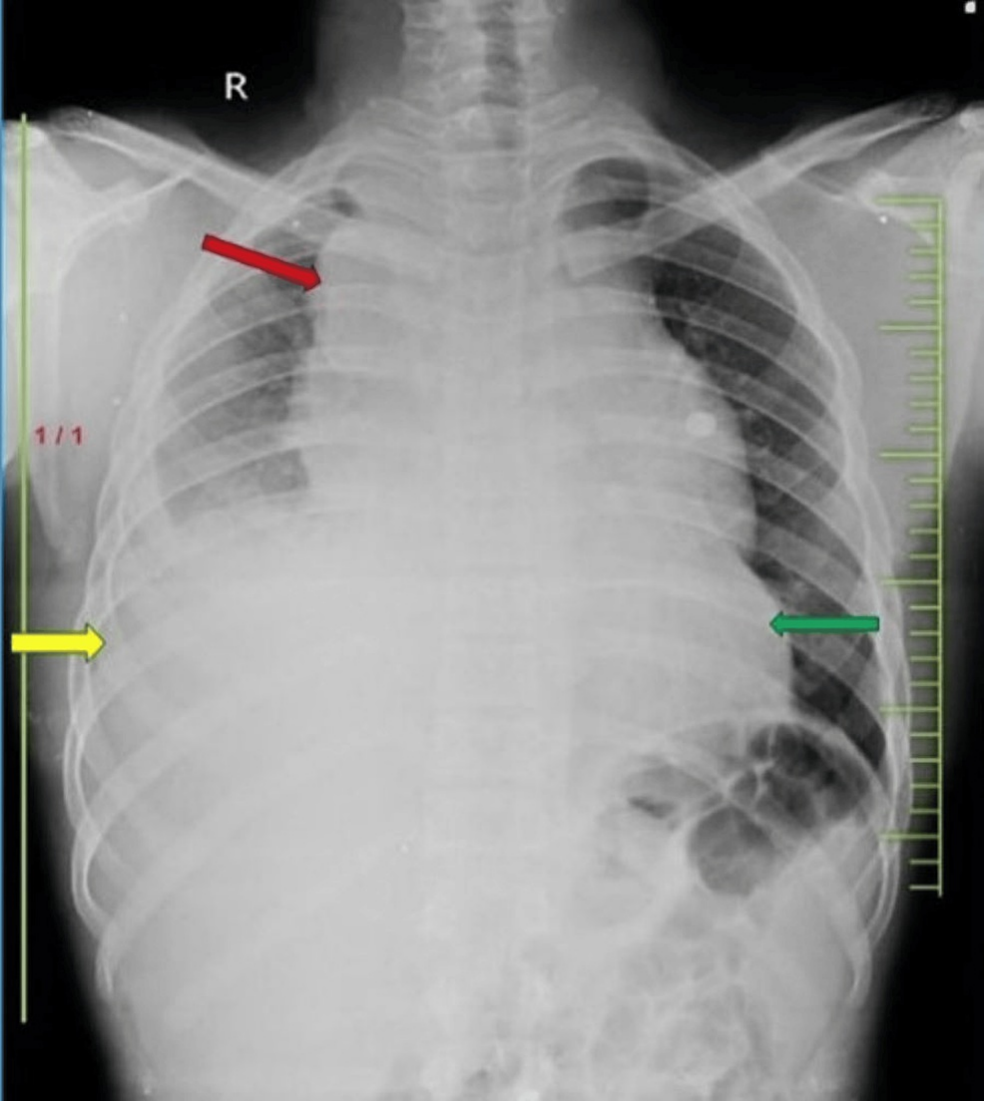
Chest X-ray showing a mediastinal mass in the right upper and middle zone extending above the clavicle (red arrow), right-sided pleural effusion obscuring the right costophrenic angle (yellow arrow), and mild cardiomegaly (green arrow).

A chest X-ray was done suggestive of radio-opacity in the right upper and middle zone extending above the clavicle suggestive of a mediastinal mass, pleural effusion on the right obscuring the right costophrenic angle, and mild cardiomegaly as shown in Figure 3.

**Figure 4:**
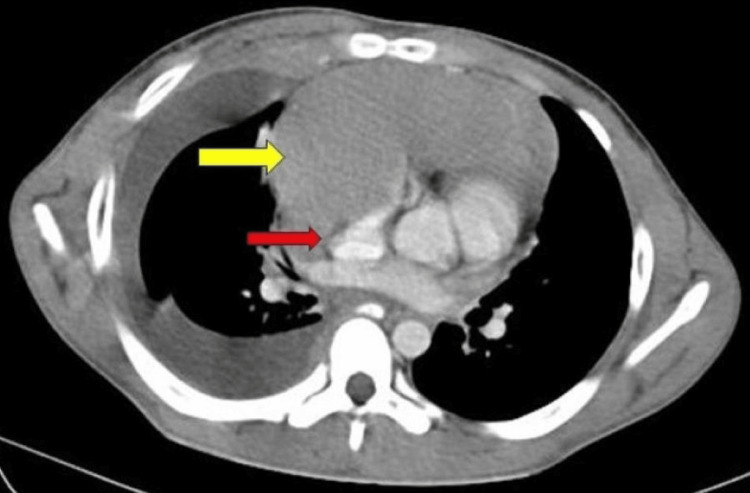
Contrast-enhanced computed tomography of the thorax showing mediastinal mass (yellow arrow) compressing the superior vena cava (red arrow).

A contrast-enhanced computed tomography of the thorax was done suggestive of a large, well-defined, homogenous anterior, and superior mediastinal solid mass measuring 19.8x10.6x13.89 with right pleural involvement, encasing vessels like the superior vena cava, azygous vein, brachiocephalic trunk, and ascending aorta, right bronchus, and trachea, anterolaterally displacing the superior vena cava causing compression and narrowing. There are right gross pleural effusion and mild pericardial effusion with a shift towards the left side as shown in Figure 4.

**Figure 5:**
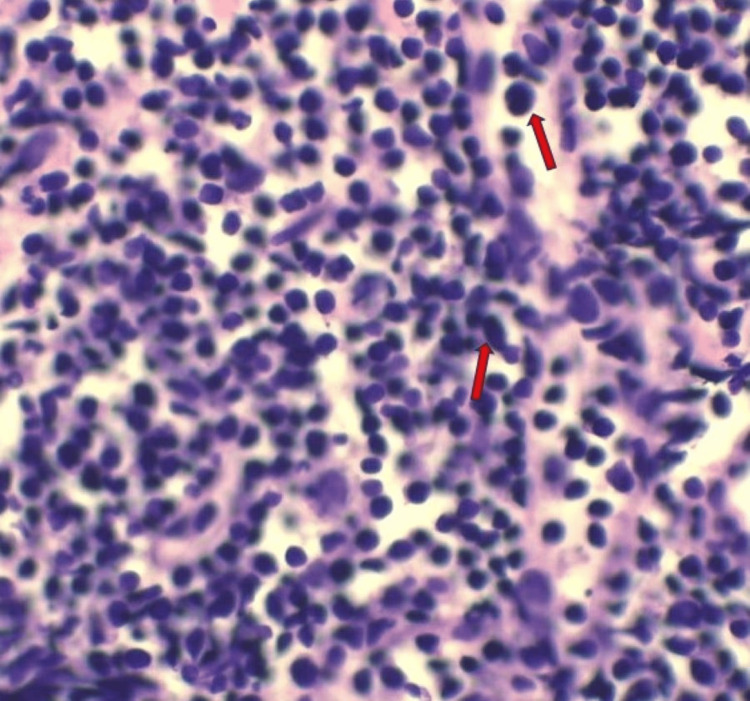
Hematoxylin and eosin-stained slide of the biopsy tissue showing sheets of lymphoid cells.

**Figure 6:**
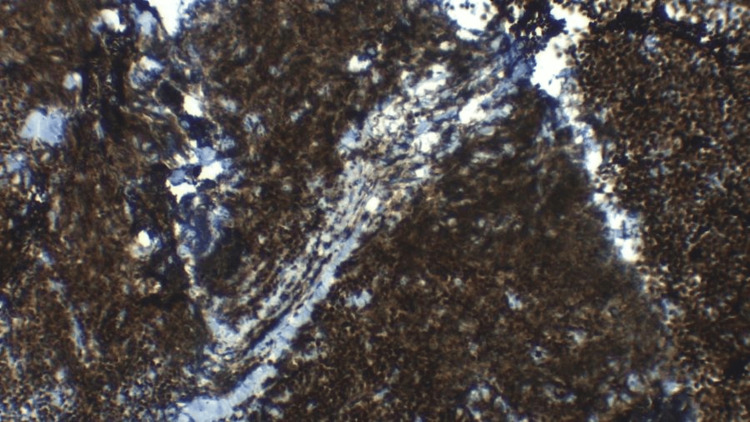
Immunohistochemistry study of the biopsy tissue: TdT showing nuclear positivity suggestive of lymphoblastic lymphoma.

To confirm the diagnosis, ultrasonography (USG)-guided biopsy was done from the anterior mediastinal mass using an 18x10 semiautomatic gun, and three to four core biopsies were taken under aseptic precautions and sent for histopathologic examinations and immunohistochemistry. Histopathology showed sheets of lymphocytes and the tumor cells were positive for CD3, BCL2, CD10, and TdT and negative for CD20, NKX2.2, and CD19, as shown in Figure 5 and Figure 6. The final diagnosis of T-LBL was established.

The patient was started on supportive treatment in view of dyspnea and cough with nasal oxygen in a propped-up position along with nebulization with budesonide (0.5 mg) three times a day. After three days of admission, the patient was symptomatically better and was referred to an oncologist. He was started on chemotherapy with the Berlin-Frankfurt-Munster (BFM) regimen consisting of vincristine, daunorubicin, prednisone, asparaginase, intrathecal cytarabine, and intrathecal methotrexate. On follow-up, after six cycles of chemotherapy, the size of the mediastinal mass has reduced, and the patient has improved symptomatically.

## Discussion

Lymphoblastic lymphoma (LBL) is a rare type of NHL that originates from precursor lymphoblasts. It represents only 1-2% of all NHLs. T-LBL is a particularly rare and aggressive type of LBL that arises from precursor T cells, accounting for 90% of all LBL cases. It commonly affects adolescents and young adults and is more common in males. On the other hand, B-cell LBL accounts for the remaining cases and tends to occur at a younger age compared to T-LBL [5].

T-LBL is usually diagnosed at the advanced metastatic stage [6]. The presence of a bulky mass in the mediastinum, often accompanied by pleural and pericardial effusion, is frequently observed during imaging. The thymus and the testes and ovaries are frequently involved [6]. In about 5-10% of cases, central nervous system (CNS) involvement is evident at the time of diagnosis. The great majority of LBLs, like other NHLs, are idiopathic, autoimmune, or inflammatory disorders, caused by exposure to radiation or chemicals, whereas T-LBL cells are thought to develop from precursor thymic T cells. The 14q11-13 region, which contains the T-cell receptor (TCR)-alpha and TCR-delta genes, is the site of the most common cytogenetic abnormalities in T-LBL. Rearrangements of the TCR-beta (7q34) and TCR-gamma (7p14.1) encoding regions are also common. T-LBL may advance rapidly, demanding immediate diagnosis and treatment. Proliferating lymphoblasts infiltrate nodal and extranodal tissues, eventually reaching the CNS, in T-LBL. This can result in gross lymphadenopathy, which can induce life-threatening compression of surrounding structures such as the tracheobronchial tree and the superior vena cava in individuals with mediastinal involvement, as shown in our case [5,6].

SVCS is a condition caused by the obstruction of blood flow through the superior vena cava, which can lead to a set of symptoms and signs. Clinical symptoms of the condition include cough, shortness of breath, and orthopnea. The most common signs of the condition are swelling in the face and arms, chest and neck vein enlargement, and chest wall dilation. Both benign and malignant conditions can result in superior vena cava blockage [2]. External compression by surrounding pathologic processes, thrombosis, or intraluminal tumor cell invasion can all lead to obstruction in malignancy-related SVCS [7]. SVCS was typically infectious in etiology before the widespread clinical use of antibiotics. Nowadays, non-malignant cases arise from thrombus formation due to intravascular devices like catheters and pacemakers. Over the past few decades, the incidence of benign cases of SVCS has increased, accounting for up to 40% of all cases. SVCS is primarily caused by mediastinal malignancies, such as lung cancer, which affects up to 10% of patients with small cell lung cancer [1]. Other causes include NHL and metastatic tumors. It was previously believed that superior vena cava blockage was an emergency, but it is now known that patients with SVCS rarely experience life-threatening consequences. Since SVCS can be the first sign of an undetected tumor in up to 60% of patients presenting with symptoms of superior vena cava blockage, as in our instance, accurate diagnosis and biopsy should come before any emergency treatment approach [1].

## Conclusions

The association between T-LBL and SVCS in a 19-year-old male is an uncommon but noteworthy clinical scenario. This case report illustrates the significance of considering such complications in the context of T-LBL, emphasizing the importance of early diagnosis and appropriate management to achieve a favorable outcome. Blockage of the superior vena cava is most commonly caused by an underlying cancer. This can lead to significant morbidity and mortality. SVCS is not typically considered a medical emergency related to cancer, but it requires prompt investigation and management in patients with an unknown history of cancer. It does not affect the chances of curing the underlying malignancy and should not change the overall treatment approach. The management of SVCS involves relieving the symptoms and treating the underlying disease. This case serves as a reminder to healthcare professionals to remain vigilant and consider rare complications, as early intervention can make a substantial difference in patient prognosis.
